# Newborn Skin Maturity Medical Device Validation for Gestational Age Prediction: Clinical Trial

**DOI:** 10.2196/38727

**Published:** 2022-09-07

**Authors:** Zilma Silveira Nogueira Reis, Roberta Maia de Castro Romanelli, Rodney Nascimento Guimarães, Juliano de Souza Gaspar, Gabriela Silveira Neves, Marynea Silva do Vale, Paulo de Jesus Nader, Martha David Rocha de Moura, Gabriela Luíza Nogueira Vitral, Marconi Augusto Aguiar dos Reis, Marcia Margarida Mendonça Pereira, Patrícia Franco Marques, Silvana Salgado Nader, Augusta Luize Harff, Ludmylla de Oliveira Beleza, Maria Eduarda Canellas de Castro, Rayner Guilherme Souza, Gisele Lobo Pappa, Regina Amélia Pessoa Lopes de Aguiar

**Affiliations:** 1 Health Informatics Center Universidade Federal de Minas Gerais Belo Horizonte Brazil; 2 Department of Gynecology and Obstetrics Universidade Federal de Minas Gerais Belo Horizonte Brazil; 3 Center for Artificial Intelligence, Innovation and Health Universidade Federal de Minas Gerais Belo Horizonte Brazil; 4 Child and Adolescent Health Universidade Federal de Minas Gerais Belo Horizonte Brazil; 5 Hospital Sofia Feldman Belo Horizonte Brazil; 6 Maternal and Child Unit, University Hospital Universidade Federal do Maranhão São Luis Brazil; 7 University Hospital of Canoas Universidade Luterana do Brasil Canoas Brazil; 8 Hospital Materno Infantil de Brasília Distrito Federal Brazil; 9 Computer Science Department Universidade Federal de Minas Gerais Belo Horizonte Brazil; 10 State Health Department of Minas Gerais Belo Horizonte Brazil

**Keywords:** gestational age, prematurity, childbirth, skin physiological phenomena, machine learning, equipment and supplies, pregnancy, reproductive health, pregnant, skin, age, medical, device, newborn, baby, trimester, therapy, learning model, ultrasound

## Abstract

**Background:**

Early access to antenatal care and high-cost technologies for pregnancy dating challenge early neonatal risk assessment at birth in resource-constrained settings. To overcome the absence or inaccuracy of postnatal gestational age (GA), we developed a new medical device to assess GA based on the photobiological properties of newborns’ skin and predictive models.

**Objective:**

This study aims to validate a device that uses the photobiological model of skin maturity adjusted to the clinical data to detect GA and establish its accuracy in discriminating preterm newborns.

**Methods:**

A multicenter, single-blinded, and single-arm intention-to-diagnosis clinical trial evaluated the accuracy of a novel device for the detection of GA and preterm newborns. The first-trimester ultrasound, a second comparator ultrasound, and data regarding the last menstrual period (LMP) from antenatal reports were used as references for GA at birth. The new test for validation was performed using a portable multiband reflectance photometer device that assessed the skin maturity of newborns and used machine learning models to predict GA, adjusted for birth weight and antenatal corticosteroid therapy exposure.

**Results:**

The study group comprised 702 pregnant women who gave birth to 781 newborns, of which 366 (46.9%) were preterm newborns. As the primary outcome, the GA as predicted by the new test was in line with the reference GA that was calculated by using the intraclass correlation coefficient (0.969, 95% CI 0.964-0.973). The paired difference between predicted and reference GAs was −1.34 days, with Bland-Altman limits of −21.2 to 18.4 days. As a secondary outcome, the new test achieved 66.6% (95% CI 62.9%-70.1%) agreement with the reference GA within an error of 1 week. This agreement was similar to that of comparator-LMP-GAs (64.1%, 95% CI 60.7%-67.5%). The discrimination between preterm and term newborns via the device had a similar area under the receiver operating characteristic curve (0.970, 95% CI 0.959-0.981) compared with that for comparator-LMP-GAs (0.957, 95% CI 0.941-0.974). In newborns with absent or unreliable LMPs (n=451), the intent-to-discriminate analysis showed correct preterm versus term classifications with the new test, which achieved an accuracy of 89.6% (95% CI 86.4%-92.2%), while the accuracy for comparator-LMP-GA was 69.6% (95% CI 65.3%-73.7%).

**Conclusions:**

The assessment of newborn’s skin maturity (adjusted by learning models) promises accurate pregnancy dating at birth, even without the antenatal ultrasound reference. Thus, the novel device could add value to the set of clinical parameters that direct the delivery of neonatal care in birth scenarios where GA is unknown or unreliable.

**International Registered Report Identifier (IRRID):**

RR2-10.1136/bmjopen-2018-027442

## Introduction

### Background

Being born before 37 weeks of gestation, which is preterm birth, is the leading cause of childhood mortality. The global preterm birth rate is approximately 11%, with a particularly high frequency in low- and middle-income countries, in association with maternal education, race, and ethnic origin [1]. However, adverse neonatal outcomes affect newborns unevenly according to the birth scenario and gestational age (GA) [1]. Mortality on the first day of life is 30 times higher in low- and medium-income countries than in high-income countries [2]. The first step in caring for preterm newborns is to identify them, which remains challenging in scenarios with scarce resources [3]. An accurate assessment of preterm newborns at birth can allow practical decisions regarding support, such as keeping the lungs airing, keeping the body warm, regulating metabolism and nutrition, or making decisions to transfer them to an intensive care unit, otherwise avoiding unnecessary interventions for term newborns [4]. Preterm neonates are more prone to death or survival with neurological sequelae. Long-term surviving preterm infants are at risk of death before the age of 5 years and at risk of presenting cognitive and motor sequelae compared with term infants [1]. The need to pinpoint early risks at birth faces the issues of reduced early access to antenatal care and a lack of access to high-cost technologies for pregnancy dating, such as obstetric echography in resource-constrained settings [5].

Some pregnancy-dating troubles arise from antenatal care. Government policies and best practices advise pregnant women to plan pregnancy to include early access to antenatal care for pregnancies to be safely monitored until birth [6]. However, many barriers to covering all pregnancies and births with due care have not been overcome, particularly in scenarios lacking well-equipped facilities [7]. Early obstetric ultrasound currently offers the best method for the establishment of GA [8]. However, lack of access to high-cost equipment, poor training, lack of skills of health professionals, and delayed antenatal care limit pregnancy dating and, consequently, detection of prematurity [5,9]. In addition, GA calculation based on the last menstrual period (LMP) is affected by memory bias, hormone-based contraception, and breastfeeding [10]. After-birth approaches for pregnancy dating, which are also extensively used, rely on professional skills for physical and neurological maturity assessment. Nevertheless, maturity scores have failed in terms of reproducibility and accuracy [3]. Meanwhile, birth weight is a helpful predictor of risk to the newborn and not GA, as size at birth results from the dynamic process of past intrauterine growth beyond the gestation length [9,11].

Reliable pregnancy dating has an impact on measuring the global burden of preterm birth and the associated risks [3,12]. Improving preterm birth outcomes requires accurate assessment of GA to instruct timely decision-making regarding neonatal care [10]. Approaches for the enhancement of the accuracy of pregnancy dating through more accurate and accessible technologies can improve pregnancy outcomes and neonatal survival rates [8,13]. Health technology development is critical for supporting health care systems. Medical devices and digital health technologies have brought innovative solutions with the potential to save lives [14], mitigating quality gaps among disparate health care scenarios [15]. Furthermore, digital health technologies have the potential to impact the equality of health care, creating new landscapes of opportunities, such as application of data science to improve prediction models [16]. Currently, computer science has advanced, with improvements to medical practice, detecting patterns by processing data sets through layered mathematical models [17], and fostering the skills and competences of professionals in support of the best health care decisions [14].

The new test explored in this study is an innovative approach used to estimate GA based on the photobiological properties of the newborn’s skin and by learning predictive models enhanced with clinical variables [18]. It being usable as a medical device, we developed this technology to easily assist health professionals in the care of newborns whenever the pregnancy dating is unknown or doubtful, adding relevant information for classification and better management of the newborn.

### Objective

This study aimed to validate a new medical device used to assess GA through the photobiological model of skin maturity adjusted to clinical data and to determine its accuracy in detecting preterm newborns. We tested the hypothesis of equivalence between GA measured by this new test and by pregnancy-dating comparators calculated using ultrasound examinations and the LMP.

## Methods

### Study Design and Participants

This study was a multicenter, prospective, intention-to-diagnosis clinical trial investigation with a single group, single-blinded, and single-arm, using a reference standard. This paper adheres to the Transparent Reporting of a Multivariable Prediction Model for Individual Prediction or Diagnosis for completeness and clarity [19]. Intention-to-diagnosis is a method for prospective studies in which all participants are considered in the statistical analysis, allowing us to reach unbiased conclusions regarding the effectiveness of an intervention [20]. To assess the risk of bias and applicability, the development and validation methods followed guidance from the Prediction Model Risk of Bias Assessment Tool [21]. The clinical trial protocol was disclosed in the World Health Organization’s International Clinical Trial Platform—Brazilian Clinical Trials (registered under trial number RBR-3f5bm5).

This report examined the primary and secondary outcomes of data concerning GA prediction and clinical safety of the novel device. Secondary outcomes related to lung maturity prediction are currently under analysis for further publication. The following five Brazilian urban referral centers for high-complexity perinatal care took part in the study: Clinical Hospital—Universidade Federal de Minas Gerais (as coordinator), Minas Gerais State; Sofia Feldman Hospital—Minas Gerais State; Hospital da Universidade Luterana do Brasil—Rio Grande do Sul State; Hospital Materno-infantil de Brasília—Federal District; and Hospital Universitário da Universidade Federal do Maranhão—Maranhão State.

A prospective concurrent and sequential process enrolled newborns during the first 24 hours of life. The first enrollment occurred on January 2, 2019, and the last occurred on May 30, 2021. Eligibility criteria, participants’ timeline, and procedures followed the research clinical protocol [22]. In short, we assessed the skin maturity of live newborns with at least ≥24 weeks of GA. All had reports of antenatal ultrasound, one from 7 to 13 weeks and 6 days and the other from 14 to 23 weeks and 6 days of gestation. Anhydramnios, hydrops, congenital skin diseases, or chorioamnionitis were the exclusion criteria, owing to their potential to modify the skin structure.

### Procedures

The coordinating unit trained 15 health professional examiners following good clinical practice as set forth by the Brazilian Regulatory Health Agency’s recommendations. Standard operating procedures were mandatory to guide the enrollment process, skin assessment, and data collection [22]. Clinical information was collected through structured questionnaires, using a software program dedicated to this project. The framework of the clinical variables and skin acquisitions is available in Multimedia Appendix 1. Textual information was saved on a tablet with internet access, individually associated with the respective skin assessment acquired using the medical device [23].

An automated algorithm in the data collection system [24] blinded to the examiner calculated the reference GA. Established rules for redating GA at birth provided our reference for GA using data from the ultrasound reports or antenatal care books or other clinical document [8]. For data curation, the investigator’s data entries were confronted with information from photographed digital images of clinical documents. In the case of multiple birth gestations with different ultrasonographic crown-rump length values, the average of each embryo or fetal value was considered. A double-check system, paper-based and electronic, allowed verification of the reliability and validity of clinical data as well as skin reflectance acquisition. In addition, the data quality of antenatal pregnancy dating was evaluated by comparing the frequency of days in dates of LMP, as they should be random with no preference for digits. For this purpose, in cases of multiple gestations, we retained only the first twin information for the day digit evaluation.

### Intervention

The intervention in this clinical trial was a test performed with a novel device that processes the backscattered signal acquired from the skin of the newborn’s sole with clinical variables to predict the GA. Its development includes steps from the workbench to clinical experimentation, as described earlier [18]. Similarly, we previously analyzed the best body position to assess skin reflectance for pregnancy dating and environmental influences such as humidity, temperature, ambient light, and the newborn’s skin hue [18,25]. Regarding the characteristics of the components, wavelengths from 400 nm to 1200 nm of the light emitter placed the safety level of this medical device in class II (noninvasive and medium risk), according to the regulatory agency in Brazil. When the light-emitting sensor touches the skin of the sole for a few seconds, it triggers 10 automated measurements. The device-emitted error warning signals were caused by the involuntary movement of the newborn or examiner under the input of ambient light by the sensor; these were events that required a new attempt [18]. The device output was blinded to the examiners. The reliability of skin reflection acquisition was assessed during the certification visit of a senior researcher in the collaborating units (Multimedia Appendix 2 [26]).

**Figure 1:**
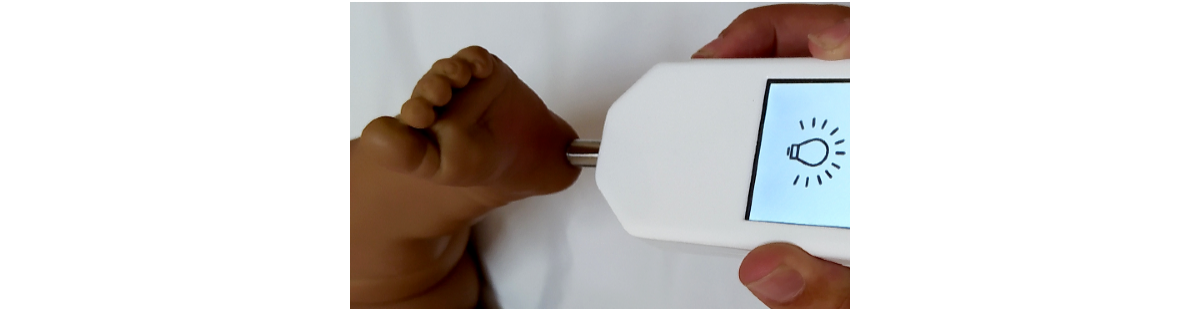
The new device and its simulated application on a newborn doll.

Skin assessments occurred with the newborn inside incubators, incubators-radiant warmers, warming pad-bassinets, standard cribs, or in the mother’s lap to ensure minimum manipulation and to avoid unbalancing the clinical conditions. The sensor touched the sole 3 times, following complete disinfection with alcohol. A total of 14 minimum viable products were produced in this study (Figure 1). At the beginning and end of the clinical trial, the irradiance emitted by each device and the reflection against a standard white wavelength calibration standard provided values for calibration. The adjusted value was the raw value of the acquisition divided by the irradiance of the light-emitting diode of each device.

**Figure 2:**
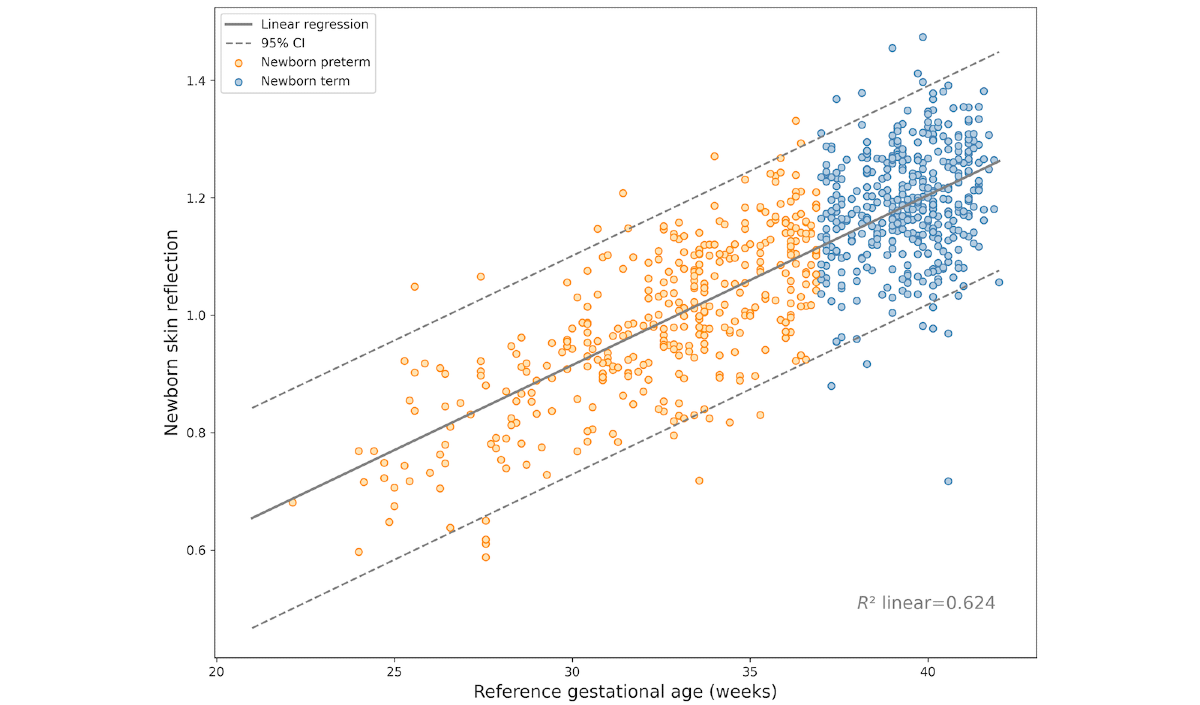
Correlation plot between the skin reflectance of the newborn and the reference gestational age at birth.

The device used an algorithm to predict GA, as previously described, and was duly patented [18]. We assessed the Pearson coefficient to confirm the correlation between skin reflectance and the reference GA. Skin reflectance had a strong positive correlation with the reference GA (*r*=0.79, *P*<.001; Figure 2).

The standalone newborn skin reflectance value was adjusted for clinical variables. The current data set the groundwork for improvements in the model for prediction of GA with the use of machine learning models as part of the research protocol. The analytical pipeline is detailed in Multimedia Appendix 3 [27-29]. The nonlinear machine learning method, Extreme Gradient Boosting (XGBoost) [27], created no more than 50 trees with a maximum depth of 3. The models were validated using a 10-fold cross-validation approach repeated 30 times. Clinical variables used as predictors of the models were available at the time of testing, which is a part of the routine of care. Therefore, they can be used in real scenarios from user input into the medical device interface.

Models’ performance with different covariates, including intermediate analysis considering factors such as incubator stay, sex, and jaundice, is presented in Multimedia Appendix 4 [18]. These new tests were performed to validate the elimination of intervenient variables after technological improvements and were added to the current version of the device [18]. Skin acquisition, duly adjusted for antenatal corticosteroid therapy for fetal maturation (ACTFM) exposure information, achieved a coefficient of determination, *R*^2^ of 0.732 and a mean absolute error (MAE) of 1.688 weeks (11.8 days). In addition, considering birth weight, the model achieved an even better performance in terms of *R*^2^ of 0.878, with an MAE of 1.147 weeks (8.0 days). This new model, with 3 predictive variables, was the one validated in this study. However, 3 GA predictions had ACTFM data imputation by the machine learning model owing to missing information because of failures in the antenatal record available at maternity admission.

### Outcomes

The primary outcome was the agreement between the GA predicted by the device and reference GA. The secondary end point was the accuracy of the device in the identification of preterm newborns, considering thresholds at 37, 32, and 28 weeks of pregnancy. Moreover, the proportion of preterm newborns correctly detected at birth within a 1-week error margin. Another secondary end point was the comparison of differences between predicted GA and GA calculated by a second ultrasound examination after 13 weeks and 6 days of gestation and before 22 weeks via comparator-ultrasound-GA and with the comparator-LMP-GA. This outcome was intended to simulate the performance of the device in scenarios without the reference and to compare the agreement between the established methods for GA calculation and the new test. The safety of the device is still a derived end point which refers to the reporting of unexpected medical events, unintended illness or injury, or adverse clinical signs in newborns, users, or others, regardless of whether they are related to the investigated product. The users answered 9 questions regarding issues with the medical device, after each skin acquisition (Multimedia Appendix 1).

### Statistical Analysis

Descriptive analyses of the newborn’s clinical characteristics and intervention measurements were performed. Regarding the primary end point, the agreement among different methods for GA at birth determination was calculated using the intraclass coefficient (ICC) correlation, Bland-Altman intervals, and the paired day difference to reference GA. Regarding the accuracy of the predicted GA by the device in identifying premature newborns, the area under the receiver operating characteristic curve (AUROC) at a CI of 95% described the new test’s discrimination and diagnostic parameters. The chi-square test, Mann-Whitney *U* test, and mean paired differences were used to compare interest groups of preterm and term newborns. *P* values of <.05 were considered suggestive of statistical significance. SPSS software (version 19.0; IBM Corporation) was used for statistical analysis of the data.

### Ethics Approval

The local independent ethics review board approved the research protocol, registered under the number CAAE 81347817.6.1001.5149 at the Brazilian National Research Council. In addition, parents signed an informed consent form on behalf of the newborns before participating.

## Results

### Study Design and Participants

**Figure 3:**
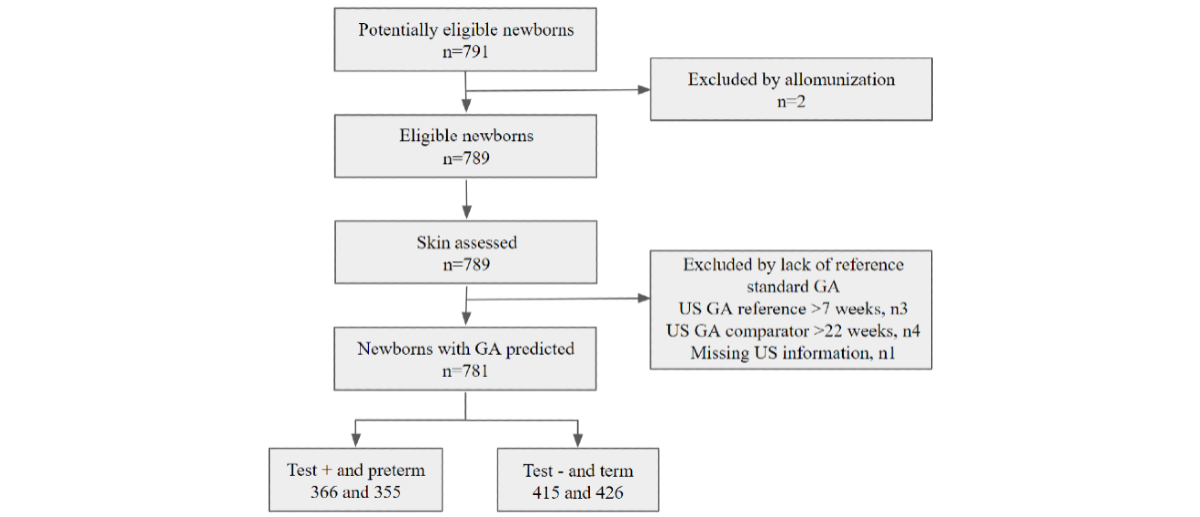
Flow diagram of participants throughout the study with results for the predictive model. GA: gestational age; US: ultrasound.

Of the 791 potentially eligible newborns, 2 were under Rh alloimmunization during pregnancy, which was considered an exclusion criterion (Figure 3). In this figure, the test is the prediction of GA with the device using the XGBoost algorithm, which includes skin reflectance, birth weight, and ACTFM exposure predictors. The positive sign (“+”) represents preterm and negative sign (“−”) represents term. Among the 789 newborns who had their skin assessed with the optical probe of the device, 8 had no reference standard to assess the dependent variable, 4 had no antenatal first-trimester ultrasound, 3 had no comparator ultrasound, and 1 had an unsolved digit date error. All 781 newborns who met the eligibility criteria for the clinical trial were included in the analysis.

The study group comprised 702 pregnant women who gave birth to 781 newborns. Despite early access to antenatal care with a median value of 12 (IQR 4) weeks (Table 1), only 296 (42.2%) women met the criteria for reliable LMP among 613 who were able to provide such a date. According to the reference GA at birth, 53.1% (415/781) of newborns were born at term. Among 366 (46.9%) preterm newborns, 235 (30.1%) had a GA at birth of 32 to 37 weeks, 131 (16.8%) had a GA of 28 to 32 weeks, and 42 (5.4%) had a GA of less than 28 weeks. Some newborns (273/781, 35.1%) received ACTFM following local protocols, and in 3 (0.4%), the data were missing. The frequency of abnormal fetal growth classification at birth was 115 (14.7%) in the small for GA group and 59 (7.6%) in the large for GA group. Approximately one-third (280/781, 35.9%) of the newborns were in the intensive care unit at the time of skin assessment.

**Table 1:** Baseline characteristics of the pregnancies and newborns.

Characteristics		Values, n	Statistics
**Maternal data**		702	N/A^a^
	Maternal age (years), median (IQR)	702	27 (9)
	First antenatal care assessment (weeks), median (IQR)	616	12 (4)
	Absent recall of last menstrual period, n (%)	702	89 (12.7)
	Reliable last menstrual period, n (%)	613	296 (42.2)
	Diabetes, n (%)	701	103 (14.7)
	Hypertensive disturbance during pregnancy, n (%)	702	1103 (14.7)
	ACTFM^b^, n (%)	698	273 (35.1)
	Multiple gestation, n (%)	702	74 (10.5)
**Neonatal data**		781	N/A
	Reference gestational age at birth (weeks), median (IQR)	781	37.3 (6.3)
	Gestational age at the first ultrasound assessment (weeks), median (IQR)	781	10.1 (3.6)
	Gestational age at the second ultrasound assessment (weeks), median (IQR)	781	19.4 (4.3)
	ACTFM exposure, n (%)	777	273 (35.1)
	Major malformation, n (%)	781	8 (1.1)
	1-min Apgar score, median (IQR)	775	8 (1)
	5-min Apgar score, median (IQR)	777	9 (1)
	Birth weight (g), median (IQR)	781	2740 (1498)
	Sex (male), n (%)	781	390 (49.9)
	Incubator accommodation at skin assessment, n (%)	781	239 (30.6)
	NICU^c^ at skin assessment, n (%)	781	280 (35.9)
	Jaundice at skin assessment, n (%)	779	255 (32.7)
	Phototherapy at skin assessment, n (%)	774	32 (4.1)
	Newborn mortality within first 72 hours, n (%)	781	14 (1.8)
	Respiratory distress syndrome until 72 hours, n (%)	781	215 (27.5)
**Classifications of newborns based on reference gestational age**			
	Preterm^d^, n (%)	781	366 (46.9)
	Moderate to late preterm^e^, n (%)	781	235 (30.2)
	Very preterm^f^, n (%)	781	89 (11.4)
	Extremely preterm^g^, n (%)	781	42 (5.4)
	Small for gestational age, n (%)	781	115 (14.7)
	Appropriate for gestational age, n (%)	781	607 (77.7)
	Large for gestational age, n (%)	781	59 (7.6)

^a^N/A: not applicable.

^b^ACTFM: antenatal corticosteroid therapy for fetal maturation.

^c^NICU: neonatal intensive care unit.

^d^Less than 37 weeks.

^e^More than 32 to less than 37 weeks.

^f^More than 28 to less than 32 weeks.

^g^Less than 28 weeks.

### Procedures: GA at Birth by Established Methods

**Figure 4:**
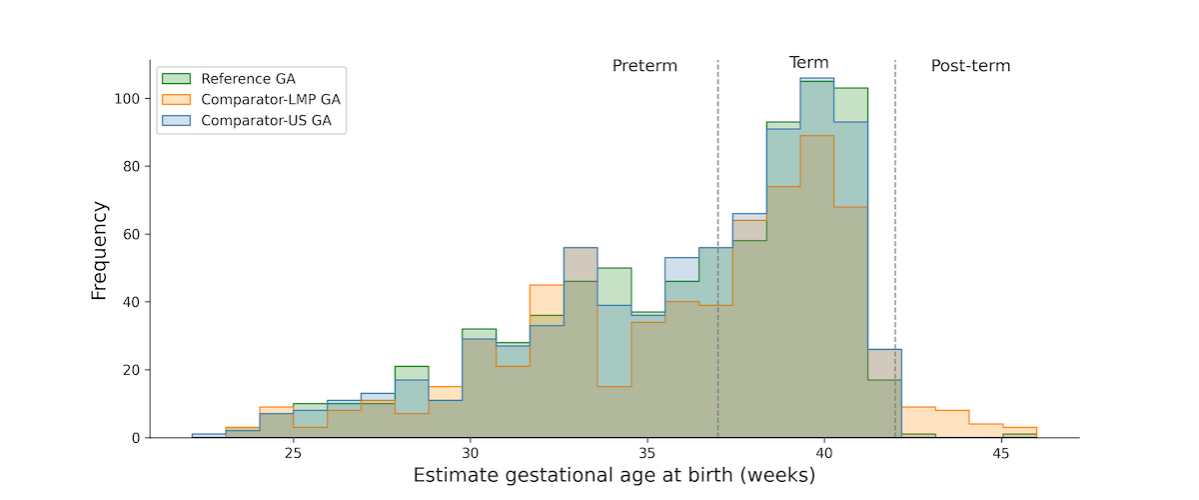
The distribution of estimated gestational age at birth by the established methods evaluated in this study. GA: gestational age; LMP: last menstrual period; US: ultrasound.

The distribution of GA as calculated according to the different references corroborated some differences among the established methods of antenatal dating, as shown in the overlapped histogram, in weeks of gestation (Figure 4). In this figure, the red dotted line corresponds to the limit between preterm and term newborns. The green dotted line corresponds to the limit between term and postterm newborns. Reference GA had a median of 37.3 (IQR 6.3) weeks, above that of the comparator-ultrasound-GA, which had a median of 37.1 (IQR 6.1) weeks, *P*<.001 (paired Wilcoxon test). However, when available, the comparator-LMP-GA had a median of 37.4 (IQR 6.8) weeks, similar to the reference GA, *P*=.282 (paired Wilcoxon test). The frequency of preterm birth was 46.9% (366/781), 47.1% (368/781), and 45.6% (310/680) according to the reference GA, comparator-ultrasound-GA, and comparator-LMP-GA, respectively. The frequency of postterm birth was 0.1% (1/781), 0.3% (2/781), and 4% (27/680) with reference to GA, comparator-ultrasound-GA, and comparator-LMP-GA, respectively. On the other hand, the data quality of the LMP recall revealed that the most frequent digit preferences were for days 5 (8.3%), 15 (6.7%), 20 (7.2%), and 25 (4.7%). These frequencies had significant differences when compared with the day adjusted to the reference GA (*P*<.008; Cochran Q test for k-related samples).

Digit preference analysis searched for the tendency of round-numbered days of the menstrual period, considering digits, typically multiples of 5 and 10. This was determined by comparing the observed and expected counts for each day of a month. The Cochran Q test for k-related samples compared the LMP with the day adjusted to the reference GA. We removed duplicate data from twins, and observations on day 31 were removed during the statistical test. The dotted line corresponds to the frequency expected for each day for 30 days per month.

Analyzing the day digit of the LMP informed by the woman, the most frequent digit preferences were for days 5 (8.3%), 15 (6.7%), 20 (7.2%), and 25 (4.7%). These frequencies had significant differences when compared to the day adjusted to the reference GA (*P*=.008). Analyzing the day digit of the LMP adjusted by the second ultrasound examination performed on data after 13 weeks and 6 days of gestation and before 22 weeks (comparator ultrasound), there were no significant differences when compared with the day adjusted to the reference GA (*P*=.20).

### Primary Outcome: GA Estimation at Birth

**Figure 5:**
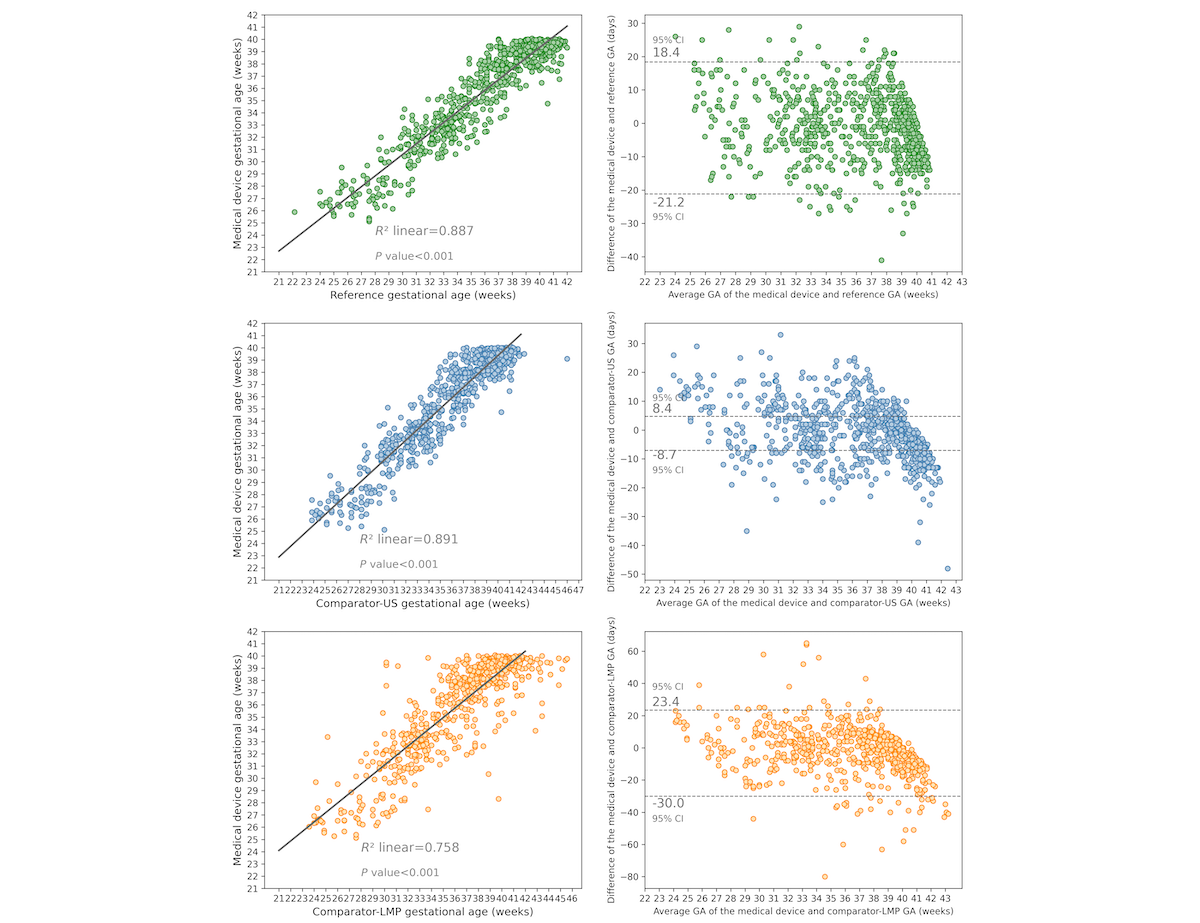
Correlation between GAs as measured using medical devices, established methods of pregnancy dating, and Bland-Altman plots. GA: gestational age; LMP: last menstrual period; US: ultrasound.

The agreement between the predicted GA, reference, and comparators was high considering the ICC (Table 2). Considering the CI of 95%, the GAs provided by the device had an ICC similar to those calculated between the reference GA and the comparator-ultrasound as well as comparator-LMP. Moreover, the ICC of predicted GA using the established methods had exceptional values (Figure 5).

The device underestimated the reference GA 1.34 (95% CI −2.04 to −0.64) days, as well as by 0.81 (95% CI −1.50 to −0.11) days, and by 2.35 (95% CI −3.49, −1.21) days in relation to the ultrasound and LMP-GA comparators, respectively. In the meantime, the ultrasound GA comparator underestimated the reference GA by −0.53 (95% CI −0.88 to −0.19) days. The end points of the Bland-Altman 95% limits of agreement were the 2.5th percentile and 97.5th percentile for the distribution of the difference between paired measurements (Figure 5). Therefore, 95% of the differences between the new test and the reference GA were within the range of −21.2 to 18.4 days. This range was shorter than that of the comparator-LMP-GA, −25.0 to 29.0, in relation to the reference GA.

**Table 2:** Agreement between predicted gestational age and the established references.

Statistic	Test^a^	*P* value	Reference GA^b,c^	*P* value
ICC^d^ with reference GA (95% CI)	0.969 (0.964 to 0.973)	N/A^e^	1	N/A
ICC with comparator-ultrasound-GA^f^ (95% CI)	0.969 (0.965 to 0.973)	N/A	0.993 (0.992 to 0.994)	N/A
ICC with comparator-LMP^g^-GA^h^ (95% CI)	0.927 (0.916, 0.938)	N/A	0.958 (0.951 to 0.964)	N/A
Day paired difference with reference GA (95% CI)	−1.34 (−2.04 to −0.64)	<.001	0	N/A
Day paired difference with comparator-ultrasound-GA (95% CI)	0−.81 (−1.50 to −.11)	<.001	−0.53 (−0.88 to −0.19)	.002
Day paired difference with LMP GA (95% CI)	−2.35 (−3.49 to −1.21)	<.001	0.83 (−0.07 to 1.74)	.071
Bland-Altman 95% limits for the medical device (days)	N/A	N/A	−21.2 to 18.4	N/A
Bland-Altman 95% limits for comparator-ultrasound (days)	−8.7 to 8.4	N/A	−10 to 8	N/A
Bland-Altman 95% limits for comparator-LMP (days)	−30 to 23.4	N/A	−25 to 29	N/A

^a^Medical device gestational age predicted using the Extreme Gradient Boosting model, based on newborn skin reflectance values, birth weight, and antenatal corticosteroid therapy for fetal maturation exposure information.

^b^GA: gestational age.

^c^Reference gestational age is the best due date.

^d^ICC: intraclass correlation coefficient.

^e^N/A: not applicable.

^f^Comparator-ultrasound-GA: gestational age calculated using a second antenatal ultrasound exam after 13 weeks and 6 days of gestation and before 22 weeks.

^g^LMP: last menstrual period.

^h^Comparator-LMP-GA: the gestational age calculated using the last menstrual period.

### Secondary Outcomes

#### GA Detection With 1-Week Error

**Figure 6:**
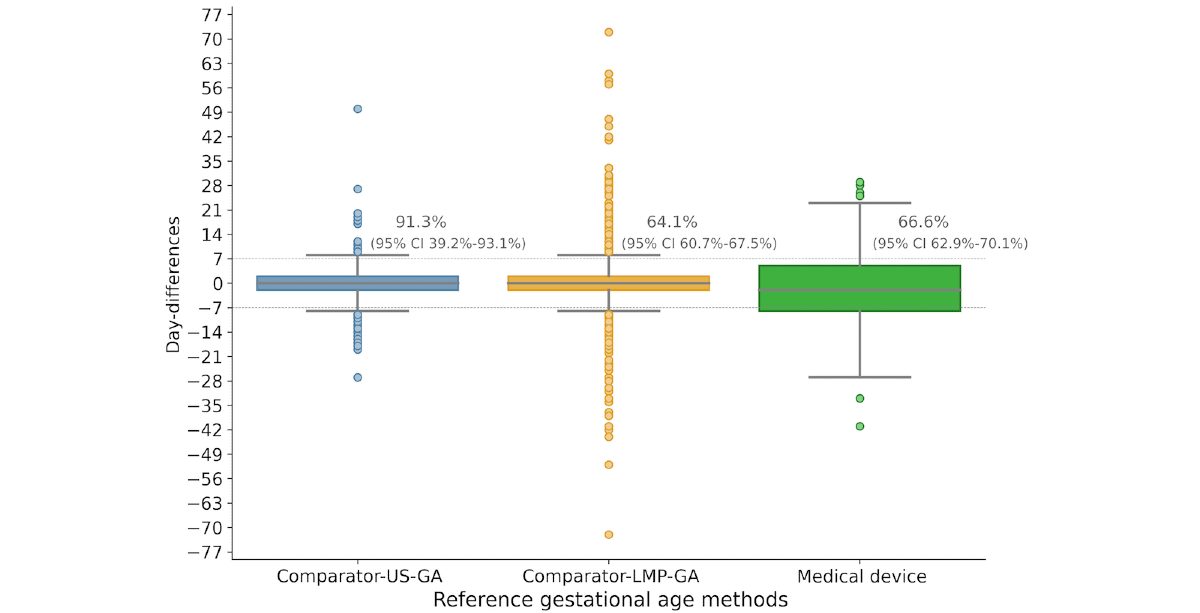
Box plot of day differences between methods and reference GA, with the proportion of agreement within 7 days. GA: gestational age; LMP: last menstrual period; US: ultrasound.

The boxplots in Figure 6 show the proportion of preterm newborns correctly detected at birth, considering an error of 1 week. We included 101 missing data points in the calculation of the rate agreement for the comparator-LMP-GA. The device achieved 66.6% (95% CI 62.9%-70.1%) of 1-week error agreement with reference pregnancy dating. This value was similar to the value of 64.1% (95% CI 60.7%-67.5%) of the comparator-LMP-GA 1-week error considering the intention-to-diagnose analysis.

#### Accuracy of the New Test for Identification of Preterm Newborns

**Figure 7:**
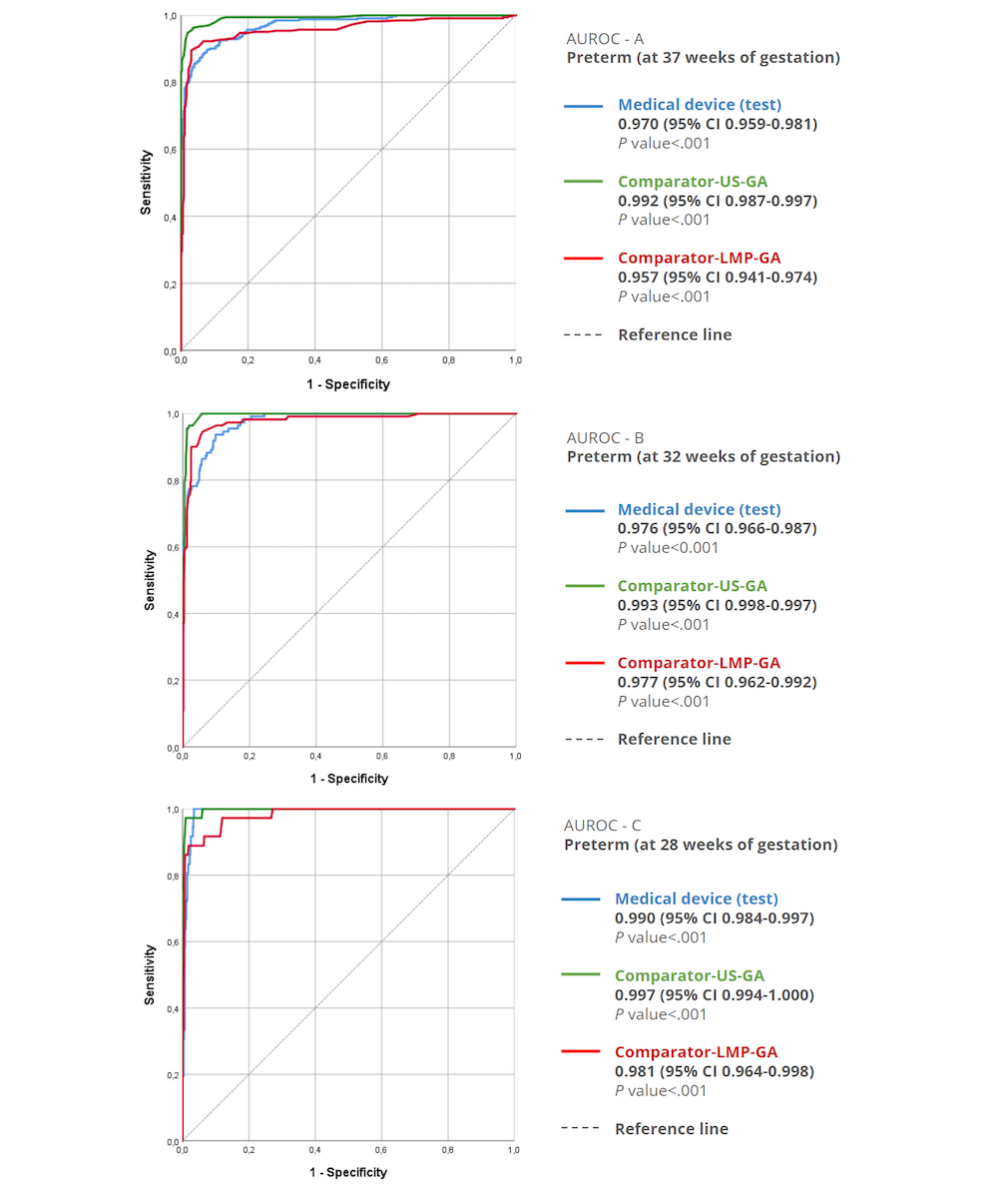
Receiver operating characteristic curves for the models to distinguish between term and preterm newborns. AUROC: area under the receiver operating characteristic curve; GA: gestational age; LMP: last menstrual period; US: ultrasound.

Considering an overlap of 95% CIs in AUROC, the new test using the device had similar performance to comparator-LMP-GA in discriminating preterm against term newborns at all cutoffs, respectively, AUROC 0.973 (95% CI 0.963-0.982) and 0.957 (95% CI 0.941-0.974; Figure 7). At cutoffs after 28 and 32 weeks, the new test had similar performance compared with the comparator-ultrasound-GA.

A comprehensive analysis of the prediction accuracy for preterm newborns using the method of GA estimation and the medical device for different prematurity cutoffs is shown in Multimedia Appendix 5. Here, we draw attention to the relevant likelihood ratio, positive at 37 weeks 13.2 (95% CI 9.2-19.0) when the medical device predicts GA, showing overlaps between the comparators in terms of 95% CI 25.0 (15.4-40.4) for comparator-ultrasound-GA and 17.1 (11.0-26.6) for comparator-LMP-GA.

#### Intent to Perform Preterm Newborn Discrimination by the Device

Birth care settings, where the device is to be applied, deserve an intent to perform preterm newborn discriminant analysis, simulating the existence of baseline references for GA calculation. Therefore, we considered newborns whose mothers had no recollection of LMP or unreliable information as scenario 1, corresponding to 451 (57.7%) newborns. In scenario 2, we grouped the newborns whose mothers had reliable LMP (Table 3). Concerning missing data, 3 test values for GA obtained using ACTFM machine learning imputation were valid results for this analysis. At the same time, 101 missing data items for LMP were newborns who had no comparator-LMP-GA owing to unknown menstrual dates.

The lack of a reliable LMP in scenario 1 resulted in low discrimination accuracy of 69.6% (95% CI 65.3%-73.7%) with the comparator-LMP-GA. Nevertheless, 89.6% (95% CI 86.4%-93.1%) of the newborns were correctly classified as preterm or term using the device. Great accuracy using any available method for GA estimation was observed in scenario 2, where the LMP was reliable. In such a scenario, we see similar device accuracy of 93.9% (95% CI 90.8%-96.3%) when compared with the accuracy of comparator-ultrasound-GA of 97% (95% CI 94.5%-98.5%) and comparator-LMP-GA of 93.4% (95% CI 94.5%-97.9%). The overall analysis includes crosstabs in Multimedia Appendix 6.

**Table 3:** Intent to perform preterm newborn discrimination according to simulated scenarios (N=781).

	Scenario 1: absent or unreliable LMP^a^ (n=451, 57.7%)					Scenario 2: reliable LMP (n=330, 42.3%)			
	Preterm newborns	Sens^b^, n/N; (95% CI)	Spec^c^, n/N; (95% CI)	ACU^d^, n/N; (95% CI)	Preterm newborns		Sens, n/N; (95% CI)	Spec, n/N; (95% CI)	ACU, n/N; (95% CI)
Reference GA^e,f^ (n=781)	199	N/A^g^	N/A	N/A	167		N/A	N/A	N/A
Test, medical device (n=781)	196	174/199; 87.4% (82%-91.7%)	230/252; 91.3% (87.1%-94.5%)	404/451; 89.6% (86.4%-92.2%)	159		153/167; 91.6% (86.3%-95.3)	157/163; 96.3% (92.2%-98.6%)	310/330; 93.9% (90.8%-96.3%)
Comparator-ultrasound-GA^h^ (n=781)	199	190/199; 95.5% (91.6%-97.9%)	241/252; 95.6% (92.3%-97.8%)	431/451; 95.6% (93.2%-97.3%)	167		162/167; 97% (93.2%-99%)	158/163; 96.9% (93%-99%)	320/330; 97% (94.5%-98.5%)
Comparator-LMP-GA^i^ (n=680)	154	131/199; 65.8% (59.1%-72.2%)	183/252; 72.6% (66.9%-77.9%)	314/451; 69.6% (65.3%-73.7%)	167		160/167; 95.8% (91.6%-98.3%)	157/163; 96.3% (93%-99%)	317/330; 93.4% (94.5%-97.9%)

^a^LMP: last menstrual period.

^b^Sens: sensitivity.

^c^Spec: specificity.

^d^ACU: accuracy (newborn correctly classified).

^e^GA: gestational age.

^f^Reference GA: is the best due date.

^g^N/A: not applicable.

^h^Comparator-ultrasound-GA: gestational age calculated using a second antenatal ultrasound exam after 13 weeks and 6 days of gestation and before 22 weeks.

^i^Comparator-LMP-GA: the gestational age calculated using the last menstrual period.

#### Safety of the Device

There were no reports of unexpected medical events, unintended illness or injury, or unfortunate clinical signs in subjects, users, or others related to the investigational product. Two devices were replaced because of an unintentional drop.

## Discussion

### Principal Findings

The main contribution of this clinical trial is the validation of a new approach for GA estimation, regardless of fetal ultrasound measures by demonstrating accurate outcomes. Based on birth weight, ACTFM exposure data, and use of a handled medical device to assess skin maturity and process algorithms, 91.4% (714/781) of newborns were correctly classified. A reliable antenatal age is a prerequisite for preterm newborn classification in birth care settings and is the first step in delivering the necessary care, considering the risks of prematurity. A term newborn, together with good tonus, breathing, or crying, is an essential element to determine steps of newborn resuscitation [30]. Although that statement seems very simple, it is quite far from reality. Without certainty as to the day in the female cycle on which conception occurred, ultrasound measurement of the crown-rump length is a consensual reference for redating pregnancy estimated by the LMP [8]. This dependence on early echographic scans has deprived many pregnant women and their babies of trustable GA [10]. Such a technological gap causes even more disparities than the difference between childbirth scenarios in fully equipped facilities and those ill-equipped with scarce technology. Moreover, it can impair the correct classification of infants as premature or growth restricted [31]. Whereas the underestimation of GA by 1.34 days on average in our results could reverberate in over care of a newborn with device implementation, neglecting a newborn at risk owing to the lack of GA data is still the worst. We believe that the risks attributed to preterm infants and the benefit of early diagnosis outweigh overdiagnosis. In addition, the delivery of neonatal care at birth is based on a set of clinical parameters, including GA [32].

In this combined study covering the enhancement of the prediction model for postnatal GA and validation of the device, we believe that the application of k-fold cross-validation with the use of machine learning algorithms provided accurate predictions [33]. While large data samples are unavailable, the process of training and testing was able to estimate the performance of algorithms until we have finished other ongoing clinical trials for external validation [34]. Furthermore, the quantification of uncertainty intervals regarding the predicted GA (calculated in days) and comparisons with established references allowed the simulation of realistic scenarios for application. Besides, the CIs accompanying AUROC accuracy contributed to revealing the forecast’s limits for discriminating terms from preterm newborns at different cutoff points with clinical relevance. Such strengths are critical for ensuring the potential value of the device in facing the challenges of postnatal identification of preterm newborns [35]. Postnatal approaches for GA assessment had characteristically shown higher errors than antenatal approaches [36]; however, studies using first-trimester ultrasound as the standard for postnatal GA comparisons were uncommon until recently. In a recent study comparing the accuracy of postnatal GA assessment, the New Ballard Score obtained −2.93 to 2.65 weeks of error compared with early ultrasound reference, analyzing a sample with 78.3% of preterm newborns [37]. In our study, the limits were −21.2 to 18.4 days, even though we did not compare the results from the medical device with any postnatal reference, it was a promising result.

Thus, data science algorithms have thus emerged with the aim of qualifying pregnancy dating. High-performance reports using learning models based on antenatal ultrasound predictors [38] contradistinguished meager outcomes from those using other morphometric postnatal predictors [3]. Moreover, valuable algorithms with postnatal combinations on the maturity scores of newborns are promising, even demanding special skills to apply [12]. Underqualified birth attendants represent a challenge in developing countries, further limiting the use of existing birth care solutions [39]. One advantage of our device is the skin assessment automation that notifies measurement errors caused by the movement of the newborn or examiner. Previous reports have detailed the human skin’s light-skin interaction and optical properties that benefit this technology [18,25].

The device’s predictive XGBoost algorithm used information that health professionals could quickly obtain in childbirth settings—the birth weight and the ACTFM exposure—and that could add value to the visual appearance of skin maturity. Explaining the model used during development, we have already demonstrated that the multivariate model for predicting GA, combining the skin reflection with birth weight, was better than these variables apart [18]. In this clinical trial, the choice had biological plausibility extending beyond mathematical reasons. Birth weight assessment is a universal step of primary routines in childbirth settings [6]. Meanwhile, predicting preterm birth based on birth weight when lacking a gold standard is far from a perfect solution. There is prior scientific evidence that birth weight is not sufficient to predict GA or a preterm newborn [9]. The weight at birth results from the dynamic process of past intrauterine growth beyond the gestation length [11]. Otherwise, the physical and neurological characteristics of maturity of the newborn adding value to predict GA are already extensively used and validated in the postnatal scores [36]. Meanwhile, the postnatal scores of newborn maturity, as the only method, have shown low accuracy in determining GA and identifying prematurity [36]. We combined birth weight and skin maturity adjusted to the ACTFM to predict GA, representing the clinical rationale with high *R*^2^ and low MAE, thereby avoiding the standalone model with birth weight (Multimedia Appendix 4).

In this trial, the GA estimated through using the device had great agreement with the reference GA at birth. The Bland-Altman test (95% limit) was lower than the comparator-LMP-GA. Moreover, this device could provide a GA to handle situations without ACTFM information as a potential tool in low-resource birth settings. Considering the simulated scenario with LMP either absent or unreliable (n=451 newborns), the new test had a better performance than the comparator-LMP for the estimation of GA. This result highlighted the context of use of this medical device, as the GA based on memory recall of the LMP missed 68 out of 199 preterm newborns, expressing a lower sensitivity when we applied the intent-to-discriminate analysis.

### Strengths and Limitations

Exposure to ACTFM played an uncertain role in the predictive model. Nevertheless, there was a rationale to consider its importance to adjust the skin reflection. Antenatal corticosteroids to improve newborn outcomes are an evidence-based intervention recommended for women at risk of preterm birth [32]. However, in addition to the acceleration of lung maturity, the effect of the drug occurs in other organs. The early fetal presence of receptors of corticosteroid hormone receptors in skin epithelial cells indicates that glucocorticoids may play an important role in the differentiation and development of human skin [40]. However, clinical evidence of the effect of ACTFM exposure on skin maturity remains unsubstantiated [41]. Thus, the adoption of the new test warrants caution. Thus, until proven otherwise, we consider that the importance of ACTFM exposure data to adjust the GA modeling is related to an effect on skin maturity. Even so, we cannot deny that antenatal exposure to corticoid therapy is more common in premature infants—264 (72.3%) of the preterm newborns in this study. In this respect, this regressor variable could imply a bias toward preterm newborn detection. The aforementioned ongoing study for external validation of the algorithms could further elucidate this issue because the enrollment process of newborns introduced the Mozambican birth scenario, where, unfortunately, ACTFM is not guaranteed for every woman at risk of preterm birth [34]. Furthermore, the accuracy is unknown for newborns with diseases that alter skin structure, which is an exclusion criterion in this study.

Current approaches to calculating GA are sensitive to data quality, resulting in an inappropriate classification of prematurity [9]. This study was committed to representing a realistic scenario regarding data quality, as stated in the research protocol, with data collection and curation to ensure the best reference and comparators for analysis. Before opening the blinding of the trial, a consistent process confronted data entries with digital images of the clinical documents taken during enrollment. Furthermore, dedicated software was developed exclusively for clinical trials, considering the quality and constraints of the variables. Part of the enrollment occurred during the COVID-19 pandemic, resulting in a minimal amount of missing data, such as yes or no for ACTFM) information (3/781, 0.4% of newborns). The lack of an LMP reference, antenatal care without early antenatal ultrasound, and unqualified date recollection for pregnancy dating at birth justify efforts to enhance the reliability of pregnancy dating through more accurate and accessible technologies to improve pregnancy outcomes and neonatal survival [10]. In our study, qualifying the LMP at birth with questions about memory regarding dates and menstrual cycles, and checking antenatal clinical documents at birth provided an estimation of GA to identify 160 preterm newborns among 167, when available.

Regarding the generalizability of the outcomes, this multicenter trial gathered referral perinatal units from Brazil’s northern, central, southwestern, and southern regions. This collaborative evaluation contributed to obtaining a sample of a mixed population of newborns with high miscegenation and involved 15 examiners who attended good clinical practice training. Both intraobserver and interobserver errors of the measurements were low, in line with previous results [18]. The number of preterm newborns was sufficient to analyze subcategories of prematurity as extreme preterm (n=42); however, the overall rate of preterm newborns was 46.9%, values observed in referral facilities for high-complexity perinatal care and not in the general population of Brazilian newborns [42]. Thus, such a high frequency might limit the representativeness of the results for the general population of newborns in low-complexity settings, where the prematurity rate is approximately 11% [1]. Among the 781 newborns, neonatal deaths during 72 hours of follow-up occurred in 14 (1.8%), with 12 deaths occurring in newborns with GA <28 weeks owing to complications arising from extreme prematurity. We expect to target the worst childbirth scenarios for this technology implementation [39]. In addition, the safety of this device is similar to that of other optical technologies already used in neonatal care [30].

### Conclusions

The assessment of newborn’s skin maturity adjusted by learning models promises accurate pregnancy dating at birth, even without the antenatal ultrasound reference. Identifying preterm newborns is the first step toward meeting their needs. The global rate of neonatal mortality is approximately 6700 neonatal deaths daily, mostly from preventable or treatable conditions in scenarios without adequate health care [43]. Without proper comparisons, the device had a lower error range than after-birth maturity scores. To provide future evidence, comparisons are expected based on postnatal approaches for GA estimation, such as scores of maturity and foot length, or image combinations [3]. We hope that strengthening the data sources of health care facilities with a reliable GA can help identify vulnerable newborns in situations without such information.

## Appendix

Multimedia Appendix 1 Database of clinical variables collected from each newborn and Preemie-Test skin acquisitions.

Multimedia Appendix 2 The reliability of the skin assessment with the photometer of the device.

Multimedia Appendix 3 Analytical pipeline.

Multimedia Appendix 4 Correlation between reference gestational age and predictor variables.

Multimedia Appendix 5 Accuracy for preterm newborn discrimination according to the methods of gestational age estimation.

Multimedia Appendix 6 Intent to perform preterm newborn discrimination according to simulated scenarios of care.

## Abbreviations

ACTFM: antenatal corticosteroid therapy for fetal maturation
AUROC: area under the receiver operating characteristic curve
GA: gestational age
ICC: intraclass correlation coefficient
LMP: last menstrual period
MAE: mean absolute error
XGBoost: Extreme Gradient Boosting

## Data Availability

Data are available upon reasonable request and after anonymization to allow for the sharing of data ethically and legally, thus preserving the confidentiality of the persons who participated in this study. The study protocol, statistical analysis plan, and informed consent form are available in previous publications. Standard operational procedures with detailed methods have been deposited in the protocols.io website.
