# Supplementary material for: Newborn Skin Maturity Medical Device Validation for Gestational Age Prediction: Clinical Trial
Source: J Med Internet Res. 2022 Sep 7;24(9):e38727. doi: 10.2196/38727 (PMC9494223; doi:10.2196/38727)
Supplement: Multimedia Appendix 1 [file jmir_v24i9e38727_app1.docx]

**Multimedia Appendix 1**

**Newborn skin maturity medical device validation for gestational age prediction: a clinical trial** (Reis, ZSN et al., 2022)

**Table S1: Database of clinical variables collected from each newborn**

| **Variables** | **Description** | **Types and categories** |
| --- | --- | --- |
| **Identification** | | |
| id_hospital | Hospital identification | Integer |
| id_user | Researcher Identification | Integer |
| nome_usuario | Researcher name | String |
| cod_sistema | Application registration code | Integer |
| ig_semanas | Gestational age (weeks) | Integer |
| ig_dias | Gestational Age (Days) | Integer |
| name_woman | Mother's name | String |
| age_woman | Mother's age | integer |
| prontuario_mulher | Women's medical record code | String |
| **Pregnancy and birth data** | | |
| data_parto | Delivery date and time | Datetime |
| us1_data | US1 - Date at the first ultrasound ( < 14 weeks) | date |
| us1_ig_sem | US1 - Gestational Age (GA) in weeks | integer |
| us1_ig_dias | US1 - GA (days) | integer |
| us1_cnn | US1 - CCN (mm) | integer |
| us2_data | US2 - Date at the second ultrasound | date |
| us2_ig_sem | US2 - GA (weeks) | integer |
| us2_ig_dias | US2 - GA (days) | integer |
| us2_bdp | US2 - Biparietal Diameter (BDP) | float |
| us2_femur | US2 - Femur | float |
| us2_cir_cefalica | US2 - Head circumference | float |
| us2_cir_abdominal | US2 - Abdominal circumference | float |
| dum_data | LMP - Last Menstrual Period | date |
| dum_confianca | LMP - Do you have confidence in LMP? | Yes / No |
| dum_cicloregulares | LMP - Are your menstrual cycles regular? | Yes / No |
| dum_concepcao_contracepcao | LMP - Did the current conception occur within 2 months of using hormonal contraceptives? | Yes / No |
| dum_concepcao_aborto | LMP - Did the current conception occur within 2 months of abortion or childbirth? | Yes / No |
| dum_ig_1visita | GA at the first antenatal care assessment (weeks) | integer |
| pront_altorisco | High-risk pregnancy | Yes / No |
| pront_diabetes | Diabetes | Yes / No |
| pront_hipertensao | Hypertensive disturb during pregnancy | Yes / No |
| pront_malformacao | Major malformation | Yes / No |
| pront_oligo | Oligohydramnios | Yes / No |
| pront_gemeos | Multiple gestations | Yes / No |
| pront_gemeos_ordem | Birth order of twins | integer |
| pront_outra_doencamaterna | Other maternal or fetal disease | String |
| cort_uso | Antenatal Corticosteroid Therapy for Fetal Maturation exposure (ACTFM) | Yes / No |
| cort_esquema | Corticotherapy scheme used | 2 doses / 4 doses |
| cort_doses | Number of doses ACTFM applied | integer |
| rn_sexo | Newborn sex | integer |
| rn_peso | Birthweight (g) | 300 - 6000 |
| rn_apgar1 | 1-minute Apgar score | 0 - 10 |
| rn_apgar5 | 5-minute Apgar score | 0 - 10 |
| me_local | Accommodation | Ward / NICU / Birth-room |
| me_tipo_leito | Type of bed of the neonate | Incubator / incubators-radiant warmer / common crib / other |
| condrni_uso_foto | Phototherapy at the skin assessment | Yes / No |
| **During 72h hours of life** | | |
| diag_ictericia | Jaundice | Yes / No |
| alta_condicoes | Discharge Conditions | discharge / death / internal |
| alta_data | Discharge date and time | Datetime |
| obito_data | Neonatal death date and time | Datetime |
| obito_causa | Death cause | String |
| fcg1 | Antenatal care book photo - Side A | Image |
| fcg2 | Antenatal care book photo - Side B | Image |
| fus1 | US1 - Ultrasound report photo (<14 weeks) | Image |
| fus2 | US2 - Ultrasound report photo (>=14 weeks) | Image |
| **Preemie-Test adverse events** | | |
| preemie_aquecimento | Adverse events: equipment heating | Yes / No |
| preemie_choque | Adverse events: electric shock | Yes / No |
| preemie_marcaspele | Adverse events: neonate's skin marks or irritation | Integer |
| preemie_display_nao_indica | Device: display indicates nothing | Integer |
| preemie_nao_carrega | Device: equipment not charging | Integer |
| preemie_disparo | Device: trigger not fired | Integer |
| preemie_led_nao_acende | Device base: red LED does not light | Yes / No |
| preemie_outroevento | Other adverse events | String |
| preemietest_ processid | Equipment serial number and sequential number | Integer |
| preemietest_ dt | Exam date and time | Datetime |
| preemietest_json | JSON file - Preemie-Test reflection | File |

**Table S2: Dataset of Preemie-Test skin acquisitions**

| **Variables** | **Description** | **Types and categories** |
| --- | --- | --- |
| preemietest_ processid | Equipment serial number and sequential number | Integer |
| preemietest_ dt | Exam date and time | Datetime |
| preemietest_json | JSON file - Preemie-Test reflection | File |
| Duration | Duration [1..3] | Integer |
| Temperature | Temperature [1..3] | Integer |
| Gain | Gain [1..3] | Integer |
| Integration Time | Integration Time [1..3] | Integer |
| Current | Current [1..3] | Integer |
| SetupTime | SetupTime [1..3] | Integer |
| LedOffRaw | LedOffRaw [1..3] | Integer |
| LedOffRawMedian | LedOffRawMedian [1..3] | Integer |
| LedOffRawMean | LedOffRawMean [1..3] | Integer |
| LedOffRawStdDev | LedOffRawStdDev [1..3] | Integer |
| LedOffCal | LedOffCal [1..3] | Integer |
| LedOffCalMedian | LedOffCalMedian [1..3] | Integer |
| LedOffCalMean | LedOffCalMean [1..3] | Integer |
| LedOffCalStdDev | LedOffCalStdDev [1..3] | Integer |
| LedOnRaw | LedOnRaw [1..3] | Integer |
| LedOnRawMedian | LedOnRawMedian [1..3] | Integer |
| LedOnRawMean | LedOnRawMean [1..3] | Integer |
| LedOnRawStdDev | LedOnRawStdDev [1..3] | Integer |
| LedOnCal | LedOnCal [1..3] | Integer |
| LedOnCalMedian | LedOnCalMedian [1..3] | Integer |
| LedOnCalMean | LedOnCalMean [1..3] | Integer |
| LedOnCalStdDev | LedOnCalStdDev [1..3] | Integer |
