# Supplementary material for: Newborn Skin Maturity Medical Device Validation for Gestational Age Prediction: Clinical Trial
Source: J Med Internet Res. 2022 Sep 7;24(9):e38727. doi: 10.2196/38727 (PMC9494223; doi:10.2196/38727)
Supplement: Multimedia Appendix 2 [file jmir_v24i9e38727_app2.docx]

**Multimedia Appendix 2**

**Newborn skin maturity medical device validation for gestational age prediction: a clinical trial** (Reis, ZSN et al., 2022)

**The reliability of the skin assessment with the photometer of the device**

The assessment of observer variability for the skin reflection acquisition with the novel device took into account the relative intra- and interobserver variability, taken in percentual, and accompanied by the 95% confidence interval (95% CI). ^1^

$(A-B)/[\frac{A+B}{2}]$

The calculation of intraobserver variability used 90 sequential measurements taken in the same delimited skin region from eight individual observers totalizing 720 skin assessments. The observers' differences between repeat acquisitions had an average (standard deviation) of 1.97% (1.89%), 95% CI: 1.84% to 2.11%. It means that on 95% of occasions, the difference between one observers' assessment did not differ more than 1.84% to 2.11%.

The calculation of interobserver variability used a difference from 120 pairs of measurements taken in the same delimited skin region by two observers, using four different devices selected by random. The average (standard deviation) of relative differences was 2.6% (2.55%), 95% CI: 2.1% to 3.1%. It means that on 95% of occasions, the difference between two observers' assessments did not differ more than 2.1% to 3.1%.

**References**

1. Popović, Z. B.; Thomas, J. D. Assessing observer variability: a user’s guide. *Cardiovascular diagnosis and therapy*. **7,** 317-324, (2017).
