# Supplementary material for: Newborn Skin Maturity Medical Device Validation for Gestational Age Prediction: Clinical Trial
Source: J Med Internet Res. 2022 Sep 7;24(9):e38727. doi: 10.2196/38727 (PMC9494223; doi:10.2196/38727)
Supplement: Multimedia Appendix 3 [file jmir_v24i9e38727_app3.docx]

**Multimedia Appendix 3**

**Newborn skin maturity medical device validation for gestational age prediction: a clinical trial** (Reis, ZSN et al., 2022)

**Analytical pipeline**

Data analysis was performed using a k-fold cross-validation procedure [1], with k=10, as illustrated in Figure 2. This cross-validation procedure was repeated N times, where N=30. This means we have generated 30 different predictions for each example in the original dataset.


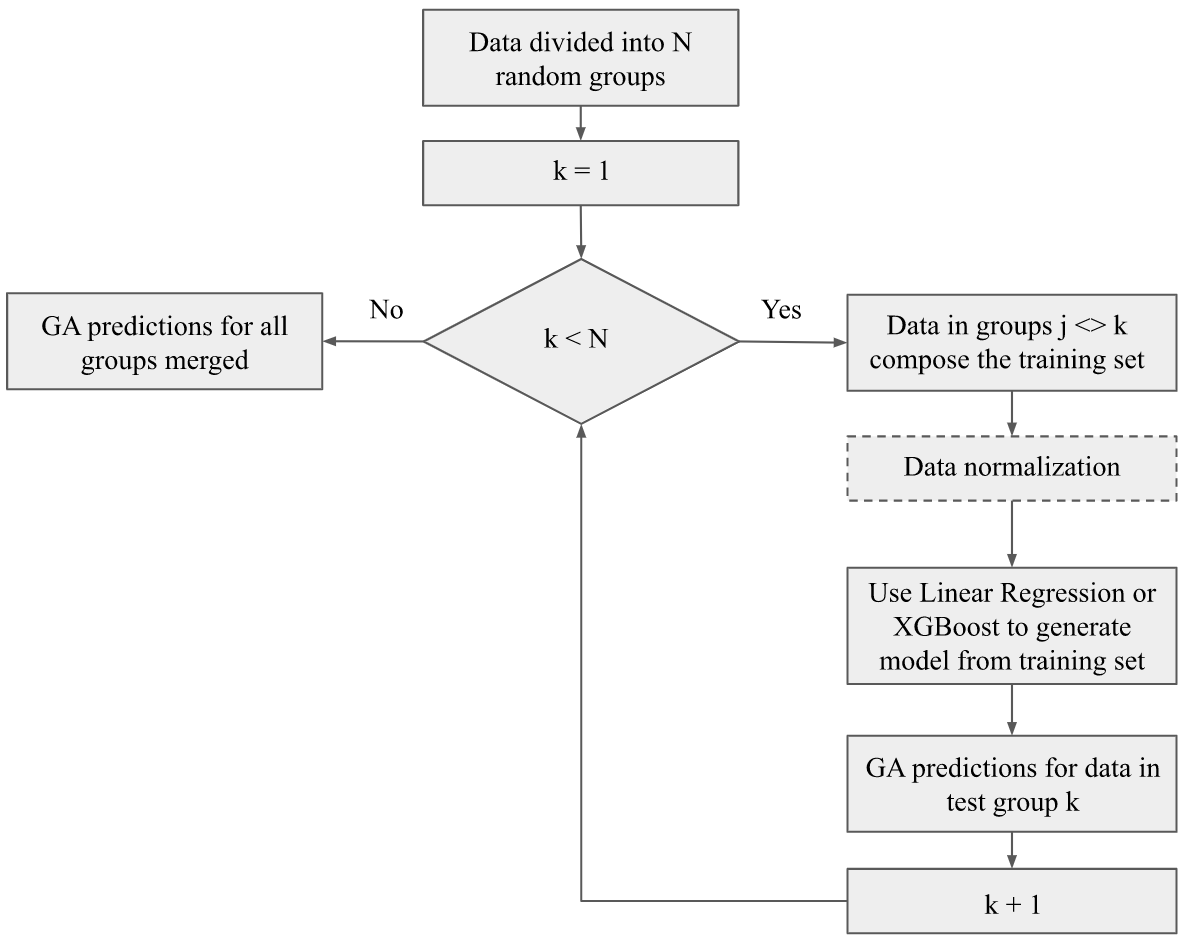


**Figure 1. Analytical pipeline**

**XGBoost**

XGBoost (Extreme Gradient Boosting) [2] is an efficient model that uses an ensemble of trees to make predictions. It combines the prediction of regression trees using the gradient tree boosting technique introduced by Friedeman [3]. The main idea behind gradient boosting is to start with a very simple model, and allow subsequent models to “correct” the residuals in the predictions generated by previous models.

XGBoost uses regression trees as base models, i.e. the simple models that will be combined. A regression tree is a binary tree, where the root and internal nodes represent an input variable and a split point (in the case of continuous attributes) into that variable. Leaf nodes contain a continuous score *w*, used to make predictions.

Consider a supervised learning scenario, where we have a dataset *D* with *n* examples and *m* features, and each example *i* is represented by a pair D = {(x_i_, y_i_)}, where xi m, yi .When we used the tree boosting method proposed by XX, the output yi of a model with *k* trees is given by:

yi = (xi) = k=1Kfk(xi),   fk F,  (1)

where F={ f(x) = wq(x)}, and q:mT, w t. *q* is the structure of the tree that maps an example to its leaf prediction (output variable), and T is the number of leaf nodes of the tree. Each fk corresponds to a tree structure *q* with leaf weights *w*. Given an example *e*, all decision trees (each represented by *q)* are used to find the corresponding leaf in the tree that classifies *e*. The final prediction is given by summing up the scores of the leaves associated with *e* in each tree (represented by *w*).

The set of functions (trees) are learnt by minimizing the regularized objective function *Obj* presented in Eq. 2.

L () = i=1nl(yi,yi)+k=1K(fk) (2)

(ft) = T + 12j=1Twj2 (3)

The first part of Eq. 2 is the loss function l(yi,yi), which measures the difference between the real value of the output variable yi and the predicted value yi. The second part is the regularization, given by the L2 norm of leaf scores, defined in Eq. 3. Its objective is to encourage leaf nodes to have smaller values (weights) and avoid overfitting.

The model described in Eq. 2 cannot be optimized using traditional optimization methods since it has functions as parameters. Hence, the model is trained in an additive way, using additive training (boosting). We start with a constant prediction and add a new function to it at each iteration, i.e., every time a new tree is generated. Let yi(t)be the prediction of the *i* instance in the *t* iteration. The process of learning the trees works as follows:

yi(0)= c

yi(1)= f1(x1) =yi(0)+f1(x1)

….

yi(t)=k=1Tfk(xi) =yi(t-1)+ft(xi)

The loss function we want to minimize can be rewritten as:

L(t)= i=1nl(yi,yi(t-1)+ft(xi))+(ft)

Observe that we add to the current model the function f_t_ that most improves the model according to Eq. 2. This loss function can be optimized using a second-order approximation. For more details on the mathematical setting, the reader is referred to Chen and Guestrin [2].

Apart from this regularized objective function, XGBoost uses the shrinkage technique to help avoid overfitting. Shrinkage scales the weights predicted by the new trees by a factor $\eta,$ known as the learning rate. It also takes advantage of feature subsampling as a third mechanism to prevent overfitting.

XGBoost can handle missing values automatically. During the training phase, for each tree node split, two paths are generated: right and left. For each path, the algorithm calculates the values of the loss function assuming the instances with missing values will be assigned to that path. The algorithm then assigns as the default direction of the node the one that leads to the smallest loss. Each node in the tree is assigned a default path, regardless of missing data for the attribute tested by that node being present on the data. During prediction, an instance with a missing value will follow the default path for a node with missing values.

**References**

1. Kohavi, R. A study of cross-validation and bootstrap for accuracy estimation and model selection. *Ijcai*. Montreal, Canada. **14**, 1137-1145 (1995).
2. Chen, T. & Guestrin, C. XGBoost: A Scalable Tree Boosting System. in *the 22nd ACM SIGKDD International Conference* 794 (2016). doi:10.1145/2939672.2939785
3. Friedman, J. H. Stochastic gradient boosting. Computational statistics & data analysis. 38, 367-378 (2002).
