# Supplementary material for: Newborn Skin Maturity Medical Device Validation for Gestational Age Prediction: Clinical Trial
Source: J Med Internet Res. 2022 Sep 7;24(9):e38727. doi: 10.2196/38727 (PMC9494223; doi:10.2196/38727)
Supplement: Multimedia Appendix 4 [file jmir_v24i9e38727_app4.docx]

**Multimedia Appendix 4**

**Newborn skin maturity medical device validation for gestational age prediction: a clinical trial** (Reis, ZSN et al., 2022)

**Correlation between reference gestational age and predictor variables**

The choice of covariates for gestational age prediction machine learning models had the following criteria: already known to be influential in pregnancy dating, or to be influential in skin reflectance phenomenon [1]. All predictors were available at the time of the test, and they could be used in real scenarios with the user input into the medical device interface: birth weight, Antenatal corticosteroid therapy for fetal lung maturity (ACTFM), incubator-stay, gender, and jaundice. The importance of such variables by the XGBoost classifier is in Figure 1.


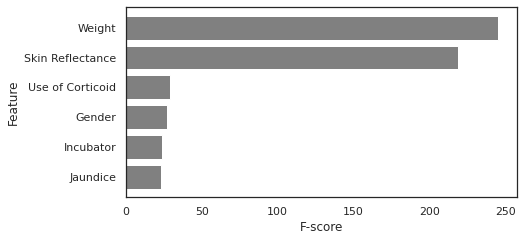


**Figure 1. The measure of feature importance returned by the XGBoost classifier**

Comparing crude variables with bivariate modeling including birth weight, mean absolute error (MAE) between predicted gestational age and reference gestational age (R) accompanied by the coefficient of determination (R^2^) highlighted the advantage on the skin reflectance adjusted to birth weight (Table 1). Concerning the models with more than two covariates to adjust the skin reflection, the coefficient of determination had differences only in the second decimal digit, being higher for the XGBoost model. MAE ranged from 1.08 to 1.16 weeks for linear regression models and 0.23 weeks with the machine learning approach, with differences in the third decimal case between models.

We choose the linear ridge regression (linear model with L2 regularization) skin reflectance algorithm as Model 1. For birth scenarios when the birth weight and ACTFM information are known, we choose the XGBOOST skin reflectance, birth weight, and ACTFM exposure as Model 2 due to the high coefficient of determination (R^2^ = 0.986) and low mean absolute error (MAE = 0.232 weeks).

**Table S3: Average results of regression models to predict R-gestational age at birth on the test sets when using a 10-fold cross-validation with 30 repetitions.**

| **Covariates used to train the models** | **Linear Regression** | | **XGBoost** | |
| --- | --- | --- | --- | --- |
|  | MAE  (SD) | R^2^  (SD) | MAE  (SD) | R^2^  (SD) |
| Birth weight (grams) | 1.309  (0.002) | 0.832  (0.002) | 1.16  (0.010) | 0·872  (0.002) |
| Skin reflectance raw model | 2.067  (0.001) | 0.612  (0.006) | 2.007  (0.009) | 0.617  (0.008 ) |
| Skin reflectance, ACTFM | 1.724  (0.002) | 0.722  (0.004) | 1.688  (0.005) | 0.732  (0.005) |
| Skin reflectance and birth weight | 1.245  (0.002) | 0.849  (0.002) | 1.278  (0.009) | 0.856  (0.003) |
| Skin reflectance, birth weight, ACTFM (n = 778) | 1.213  (0.002) | 0.856  (0.004) | 1.147  (0.009) | 0.878  (0.002) |
| Birth weight, ACTFM (n = 778) | 1.278  (0.002) | 0.841  (0.002) | 1.227  (0.007) | 0.860  (0.002) |
| Skin reflectance, birth weight, ACTFM, jaundice (n = 776) | 1.211  (0.002) | 0.858  (0.029) | 1.133  (0.006) | 0.882  (0.002) |
| Skin reflectance, birth weight, gender, jaundice, incubator (n=779) | 1.177  (0.002) | 0.867  (0.002) | 1.111  (0.007) | 0.888  (0.002) |

ACTFM: Antenatal corticosteroid therapy for fetal lung maturity. MAE: Mean absolute error (weeks). R^2^: Coefficient of determination. SD: standard deviation. For XGBoost, as it can handle missing values, N is always 781. For linear regression, N is indicated in the column “Covariates“ when different from 781.

**References**

1. Reis, Z. S. N., Vitral, G. L. N., de Souza, I. M. F., Rego, M. A. S. & Guimaraes, R. N. Newborn skin reflection: Proof of concept for a new approach for predicting gestational age at birth. A cross-sectional study. PloS One 2017. 12, e0184734.
