# Supplementary material for: Newborn Skin Maturity Medical Device Validation for Gestational Age Prediction: Clinical Trial
Source: J Med Internet Res. 2022 Sep 7;24(9):e38727. doi: 10.2196/38727 (PMC9494223; doi:10.2196/38727)
Supplement: Multimedia Appendix 5 [file jmir_v24i9e38727_app5.docx]

**Multimedia Appendix 5**

**Newborn skin maturity medical device validation for gestational age prediction: a clinical trial** (Reis, ZSN et al., 2022)

**Table S4: Accuracy for preterm newborn discrimination according to the methods of gestational age estimation**

|  | **Comparator US GA**  **(n=781)** | **Comparator LMP GA**  **(n=680)** | **Medical device - Test**  **(n=781)** |
| --- | --- | --- | --- |
| **Preterm <37 weeks (n=366)** | **Value (95% CI)** | **Value (95% CI)** | **Value (95% CI)** |
| ACU  (%) | 96.2 (94.6 to 97.4) | 92.8 (90.6 to 94.6) | 91.4 (89.2 to 93.3) |
| False - (%) | 3.8 (2.1 to 6.3) | 9.3 (6.4 to 13.1) | 10.7 (4.5 to 9.6) |
| SPE (%) | 96.1 (93.8 to 97.8) | 94.7 (91.9 to 96.8) | 93.3 (90.4 to 95.5) |
| VPP (%) | 95.7 (93.2 to 97.3) | 93.9 (90.8 to 96.0) | 92.1 (89.1 to 94.4) |
| VPN (%) | 96.6 (94.5 to 97.9) | 91.9 (89.0 to 94.1) | 90.9 (88.1 to 93.0) |
| LR+ | 25.0 (15.4 to 40.4) | 17.1 (11.0 to 26.6) | 13.24 (9.24 to 18.97) |
| LR- | 0.04 (0.02 to 0.07) | 0.10 (0.07 to 0.14) | 0.11 (0.08 to 0.15) |
| **Preterm <32 weeks (n=131)** | **Value (95% CI)** | **Value (95% CI)** | **Value (95% CI)** |
| ACU (%) | 98.5 (97.3 to 99.2) | 96.2 (94.5 to 97.5) | 92.8 (90.8 to 94.5) |
| False - (%) | 3.8 (1.3 to 8.7) | 10.0 (5.1 to 17.2) | 19.9 (13.4 to 27.7) |
| SPE (%) | 98.9 (97.8 to 99.6) | 97.4 (95.7 to 98.5) | 95.4 (93.5 to 96.9) |
| VPP (%) | 94.7 (89.6 to 97.4) | 86.8 (80.0 to 91.6) | 77.8 (71.0 to 83.4) |
| VPN (%) | 99.2 (98.2 to 99.7) | 98.1 (96.6 to 98.9) | 99.6 (94.4 to 97.1) |
| LR+ | 89.3 (42.7 to 186.8) | 34.2 (20.7 to 56.6) | 17.37 (12.12 to 24.89) |
| LR- | 0.04 (0.02 to 0.09) | 0.10 (0.06 to 0.18) | 0.21 (0.15 to 0.29) |
| **Preterm <28 weeks (n=42)** | **Value (95% CI)** | **Value (95% CI)** | **Value (95% CI)** |
| ACU (%) | 99.0 (98.0 to 99.6) | 98.5 (97.3 to 99.3) | 97.3 (95.9 to 98.3) |
| False - (%) | 11.9 (4.0 to 25.6) | 16.7 (6.4 to 32.8) | 21.4 (10.3 to 36.8) |
| SPE (%) | 99.6 (98.8 to 99.9) | 99.4 (98.4 to 99.8) | 98.4 (97.2 to 99.2) |
| VPP (%) | 92.5 (79.9 to 97.5) | 88.2 (73.6 to 95.3) | 73.3 (60.6 to 83.1) |
| VPN (%) | 99.3 (98.5 to 99.7) | 99.1 (97.3 to 99.3) | 98.8 (97.8 to 99.3) |
| LR+ | 217 (69.8 to 675) | 134 (50 to 360.3) | 48.39 (27.01 to 86.68) |
| LR- | 0.12 (0.05 to 0.27) | 0.17 (0.08 to 0.35) | 0.22 (0.12 to 0.39) |

GA: gestational age. LR+: Likelihood ratio positive. Likelihood ratio negative: LR-. SEN: sensibility. SPE: Specificity. NPV: Negative Predictive Value. PPV: Positive Predictive Value. Comparator-US GA is the gestational age calculated with a second antenatal ultrasound exam after 13 weeks and 6 days of gestation and before 22 weeks. Comparator-LMP GA is the gestational age calculated with the last menstrual period. The new test is gestational age-predicted with the XGBoost algorithm, based on newborn skin reflectance values, birth weight, and ACTFM exposure information.
