# Supplementary material for: Newborn Skin Maturity Medical Device Validation for Gestational Age Prediction: Clinical Trial
Source: J Med Internet Res. 2022 Sep 7;24(9):e38727. doi: 10.2196/38727 (PMC9494223; doi:10.2196/38727)
Supplement: Multimedia Appendix 6 [file jmir_v24i9e38727_app6.docx]

**Multimedia Appendix 6**

**Newborn skin maturity medical device validation for gestational age prediction: a clinical trial** (Reis, ZSN et al., 2022)

**Intent to preterm newborn discrimination according to simulated scenarios of care**

**Table S5: Scenario-one: Absent or unreliable LMP (n = 451, 57.7%)**

|  | Medical device (Test) | | | Comparator-US GA | | | Comparator-LMP GA | | |
| --- | --- | --- | --- | --- | --- | --- | --- | --- | --- |
|  | Preterm newborn | Term newborn | Total | Preterm newborn | Term newborn | Total | Preterm newborn | Term newborn | Total |
| Test + | 174 | 22 | 196 | 190 | 11 | 201 | 131 | 13 | 144 |
| Test - | 25 | 230 | 255 | 9 | 241 | 250 | 23 | 183 | 206 |
| Missing | 0 | 0 | 0 | 0 | 0 | 0 | 45 | 56 | 101 |
| **Total** | **199** | **252** | **451** | **199** | **252** | **451** | **199** | **252** | **451** |
|  |  | | 95% CI |  | | 95% CI |  | | 95% CI |
| Sensitivity % | 87.4 | | 82.0% to 91.7% | 95.48 | | 91.59 to 97.91 | 65.83 | | 58.79 to 72.39 |
| Specificity % | 91.3 | | 87.1% to 94.5% | 95.63 | | 92.32 to 97.80 | 72.62 | | 66.67 to 78.03 |
| Accuracy % | 89.6 | | 86.4% to 92.2% | 95.57 | | 93.23 to 97.27 | 69.62 | | 65.15 to 73.84 |
| PPV % | 88.9 | | 84.1% to 92.2% | 94.53 | | 90.64 to 96.86 | 90.97 | | 85.58 to 94.48 |
| NPV % | 90.2 | | 86.4% to 93.0% | 96.40 | | 93.39 to 98.07 | 88.83 | | 84.49 to 92.08 |
| LR+ | 10.02 | | 6.70 to 14.98 | 21.873 | | 12.26 to 39.02 | 12.83 | | 7.55 to 21.78 |
| LR- | 0.14 | | 0.10 to 0.20 | 0.047 | | 0.02 to 0.09 | 0.16 | | 0.11 to 0.23 |

Notes: CI: confidence interval. GA: gestational age. US: ultrasound. LR+: Likelihood ratio positive. Likelihood ratio negative: LR-. SEN: sensibility. SPE: Specificity. NPV: Negative Predictive Value. PPV: Positive Predictive Value. The medical device is the new test is gestational age-predicted with the XGBoost algorithm, based on newborn skin reflectance values, birth weight, and ACTFM exposure information. Comparator-US GA: the gestational age calculated with a second antenatal ultrasound exam after 13 weeks and 6 days of gestation and before 22 weeks. Comparator-LMP GA: the gestational age calculated with the last menstrual period.

**Table S6: Scenario-two: Reliable LMP (n = 330, 42.3%)**

|  | **Medical device** | | | **Comparator-US GA** | | | **Comparator-LMP GA** | | |
| --- | --- | --- | --- | --- | --- | --- | --- | --- | --- |
|  | **Preterm newborn** | **Term newborn** | **Total** | **Preterm newborn** | **Term newborn** | **Total** | **Preterm newborn** | **Term newborn** | **Total** |
| Test + | 153 | 22 | 159 | 162 | 5 | 167 | 160 | 6 | 166 |
| Test - | 14 | 157 | 171 | 5 | 158 | 163 | 7 | 157 | 164 |
| Missing | 0 | 0 | 0 | 0 | 0 | 0 | 0 | 0 | 0 |
| **Total** | **167** | **163** | **330** | **167** | **163** | **330** | **167** | **163** | **330** |
|  |  | | 95% CI |  | | 95% CI |  | | 95% CI |
| Sensitivity % | 91.62 | | 86.33% to 95.34 | 97.01 | | 93.15 to 99.02 | 95.81 | | 91.55 to 98.30 |
| Specificity % | 96.32 | | 92.16% to 98.64% | 96.93 | | 92.99 to 99.00 | 96.32 | | 92.16 to 98.64 |
| Accuracy % | 93.94 | | 90.79% to 96.26% | 96.97 | | 94.50 to 98.54 | 96.06 | | 93.36 to 97.89 |
| PPV % | 96.23 | | 92.07% to 98.25% | 97.01 | | 93.18 to 98.72 | 96.39 | | 92.40 to 98.32 |
| NPV % | 91.81 | | 87.16% to 94.88% | 96.93 | | 93.02 to 98.68 | 95.73 | | 91.56 to 97.89 |
| LR+ | 24.89 | | 11.33 to 54.66 | 31.624 | | 13.34 to 74.99 | 26.028 | | 11.86 to 57.12 |
| LR- | 0.09 | | 0.05 to 0.14 | 0.031 | | 0.01 to 0.07 | 0.044 | | 0.02 to 0.09 |

Notes: CI: confidence interval. GA: gestational age. US: ultrasound. LR+: Likelihood ratio positive. Likelihood ratio negative: LR-. SEN: sensibility. SPE: Specificity. NPV: Negative Predictive Value. PPV: Positive Predictive Value. Medical device: test using the medical device is the gestational age predicted with skin reflectance, birth weight, and ACTFM exposure predictors with the XGBoost model. Comparator-US GA: the gestational age calculated with a second antenatal ultrasound exam after 13 weeks and 6 days of gestation and before 22 weeks. Comparator-LMP GA: the gestational age calculated with the last menstrual period.
